# Bayesian network enables interpretable and state-of-the-art prediction of immunotherapy responses in cancer patients

**DOI:** 10.1093/pnasnexus/pgad133

**Published:** 2023-04-13

**Authors:** Hideki Hozumi, Hideyuki Shimizu

**Affiliations:** School of Medicine, Keio University, Tokyo 160-8582, Japan; Department of AI Systems Medicine, M&D Data Science Center, Tokyo Medical and Dental University, Tokyo 113-8510, Japan

**Keywords:** Bayesian network, tree-augmented naïve Bayes, nonsmall cell lung cancer, immune checkpoint inhibitors

## Abstract

Immune checkpoint inhibitors, especially PD-1/PD-L1 blockade, have revolutionized cancer treatment and brought tremendous benefits to patients who otherwise would have had a limited prognosis. Nonetheless, only a small fraction of patients respond to immunotherapy, and the costs and side effects of immune checkpoint inhibitors cannot be ignored. With the advent of machine and deep learning, clinical and genetic data have been used to stratify patient responses to immunotherapy. Unfortunately, these approaches have typically been “black-box” methods that are unable to explain their predictions, thereby hindering their responsible clinical application. Herein, we developed a “white-box” Bayesian network model that achieves accurate and interpretable predictions of immunotherapy responses against nonsmall cell lung cancer (NSCLC). This tree-augmented naïve Bayes (TAN) model accurately predicted durable clinical benefits and distinguished two clinically significant subgroups with distinct prognoses. Furthermore, our state-of-the-art white-box TAN approach achieved greater accuracy than previous methods. We hope that our model will guide clinicians in selecting NSCLC patients who truly require immunotherapy and expect our approach to be easily applied to other types of cancer.

**Significance Statement**

Immune checkpoint inhibitors have revolutionized cancer treatment. Given that only a small fraction of patients respond to immunotherapy, patient stratification is a pressing concern. However, the “black-box” nature of most proposed stratification methods and their insufficient accuracy have hindered their clinical application. Here, we have developed an interpretable graphical approach that achieves an even superior predictive performance than that of cutting-edge methods, while preserving interpretability. We present an approach not previously explored, evidence of a specialized graphical “white-box” model that achieves state-of-the-art performance in immunotherapy response prediction, providing strong support for the applicability of interpretable artificial intelligence (AI) models in clinical decision-making.

## Introduction

Lung cancer is the most prevalent cancer and the leading cause of cancer-related death worldwide (1). Nonsmall cell lung cancer (NSCLC) accounts for nearly 85% of all lung cancers, and its 5-year survival rate remains dismal, ranging from 68% in patients with stage IB cancer to 10% in patients with stage IVA–IVB cancer (2). Since the invention of immune checkpoint inhibitors (ICIs), many patients have gained tremendous benefits such as improved life expectancy (3). For instance, nivolumab, an inhibitor of the programmed cell death 1 (PD-1)/ligand (PD-L1) pathway, increased the 2-year survival rate of patients with stage IIIB/IV cancer from 16% to ∼30% (4).

The decision to administer ICIs to NSCLC patients has been based primarily on the expression level of PD-L1 on the surface of cancer cells, referred to as PD-L1 score (4). In most cases, patients with higher PD-L1 scores are deemed suitable candidates for ICI treatment. Nonetheless, numerous studies have demonstrated that not all patients with higher PD-L1 scores respond to ICIs and that some patients with lower PD-L1 scores respond to ICIs (4–6). According to a meta-analysis of randomized controlled trials, PD-L1 expression alone was insufficient to predict immunotherapy response (6). Additionally, the PD-L1-based predictive ability was reported to be 0.646 [based on the area under the curve (AUC)] (7), indicating that other factors must determine immunotherapy benefits. Further, immunotherapy can have devastating side effects, particularly immune-related adverse events such as pancreatitis and interstitial pneumonia (8). Thus, the use of ICIs in patients who do not respond to treatment may eventually reduce their life expectancy. Therefore, it is urgent to elucidate the factors other than PD-L1 score that determine the response and prognosis of patients under immunotherapy (9). A previous study aimed to identify factors for the stratification of NSCLC patients on ICI treatment and focused on the tumor mutational burden (TMB). Tumors with high TMB contain many neoantigens and generally respond well to ICIs (9). However, the predictive ability of TMB was 0.601, based on AUC (7). Understandably, rather than relying on a single indicator (such as PD-L1 score or TMB) to predict immunotherapy response, methods combining multiple factors have emerged. For example, LIPI (10) and EPSILoN (11) integrate clinical data such as clinical stage, performance status, and smoking status. The ratio of neutrophils to lymphocytes, a predictor of rapid progression (9), has also been incorporated into these methods. Despite this, the prediction of immunotherapy response rate has remained inadequate, with AUC values of 0.606 and 0.666 for LIPI and EPSILoN, respectively (12). This indicates that classical approaches cannot provide satisfactory predictions concerning immunotherapy.

In recent years, machine learning (ML) methods have been applied to unravel the factors determining the efficacy of ICI treatment for NSCLC. For one example, the AUC of a neural network (NN) model that integrated several factors (TMB, PD-L1 score, mutant allele tumor heterogeneity, and immune-related pathways) was as high as 0.80 in a test cohort (13). Another study integrating PD-L1 score and CT images achieved an AUC of 0.76 (14). Other ML methods, such as LightGBM, XGBoost, and regression analysis, have also been investigated (15). Although they harness various types of information (PD-L1 score, radiological images, and clinical features) as input, the AUC remains below 0.80 even for their best models, indicating that predicting responses in ICI therapy remains challenging. Moreover, ML methods, including NNs, often lack transparency due to the complexity arising from neural connections and mathematical abstractions (16–18), making it potentially impossible to explain their predictions. This “black-box” nature has hindered the clinical application of established models. Therefore, predictive models with higher accuracy and accountability are necessary for the appropriate use of ICIs in NSCLC patients.

With this in mind, we harnessed Bayesian theory and developed an interpretable artificial intelligence (AI) model with state-of-the-art predictive power in immunotherapy. Specifically, we utilized Bayesian network (BN)-based models that employ causal relationships in the form of a graphical model (19), allowing us to avoid the “black-box” problems prevalent in other ML methods (16). Hence, we demonstrate that a tree-augmented naïve Bayes (TAN) model predicted the durable clinical benefit (DCB) of patients treated with ICIs with comparable or even better accuracy than that of conventional ML methods, stratifying patients in a clinically significant manner. Further, it achieved robust predictive ability even with limited information. This data-driven approach can be used to further elucidate the factors determining immunotherapeutic responses. We anticipate that our interpretable and state-of-the-art approach will expand the knowledge of immunotherapy and be readily applicable to other types of cancer.

## Results

### Manual curation of clinical information related to immunotherapy

**Fig. 1.**
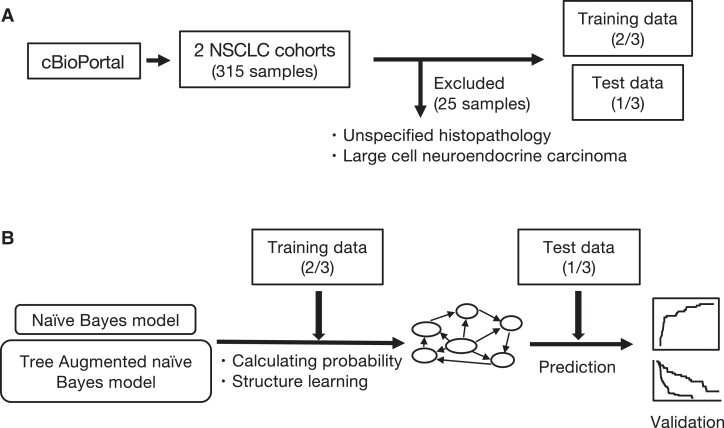
Workflow of the study. A) We obtained clinical and genetic data of NSCLC patients from cBioPortal (http://www.cbioportal.org). There were 315 data samples, of which 25 samples were excluded because they had insufficient histopathology data or because the disease was rare. Two-thirds of the data were used to construct the models (training data) and one-third, for evaluation (test data). B) We developed the NB and TAN models and evaluated their predictive accuracy for whether patients will benefit from immunotherapy. We performed survival analyses to compare the two groups based on the binary predictions of the TAN model.

To develop a state-of-the-art explanatory model, we first retrieved data for immunotherapy-receiving cancer patients from cBioPortal (http://www.cbioportal.org), which offers clinical data with information on genetic variants (20). Specifically, two previously published studies (7, 21) examining the effect of ICIs on NSCLC patients were selected and used as the data set for this study: the cohorts from Hellman et al. (21) and Rizvi et al. (7), comprising of 75 and 240 NSCLC patients who underwent immunotherapy, respectively. In total, our data set included 315 patients (Fig. 1A). The characteristics of the patients in our data set are shown in Table S1.

Among the available clinical information, we set the DCB as the objective variable, which is defined in the revised RECIST guideline (version 1.1) as partial response/stable disease lasting >6 months (22). We focused on the DCB because the follow-up criteria for overall survival and progression-free status were inconsistent between the two cohorts (7, 21). Given that the DCB has been used to assess the efficacy of immunotherapy for various tumors such as melanoma (23) and lung cancer (7), we believed that predicting the DCB was of clinical value for stratifying the patients in our study.

We used the three known clinical risk factors of NSCLC: age (<65 years of age or not) (24), sex (25), and smoking status (26). Our model also incorporated histopathological information because the pathological subtype is known to substantially affect prognosis (27). We excluded 25 samples for which there was insufficient histopathological information (Fig. 1A).

Genetic covariates were determined in two ways: first, genes in our data set with variant rates >10% were incorporated (hereafter, the “frequency-based geneset”); these included *TP53*, *KRAS*, *TTN*, *KMT2C*, *SMARCA4*, *STK11*, and *KEAP1*. Second, based on a literature survey, we identified six genes [*KRAS* (28), *STK11* (29, 30), *TP53* (31), *EGFR* (32), *ALK* (32), and *ROS1* (32)] associated with NSCLC patient ICI treatment responses or prognosis (hereafter, “evidence-based geneset”). We categorized “deletion,” “in-frame deletion,” “frameshift deletion,” “splice variant,” and “missense” modifications as “genetic variants” since they would likely impair the original function of the gene (33, 34).

We attempted to decipher the relationships underlying the DCB by combining clinical characteristics and genetic variant data. For this purpose, we randomly divided the data set into training and test data sets in a 2:1 ratio, respectively (Fig. 1A), using the former to build a model and the latter solely for evaluation (35). Receiver operating characteristic (ROC) analysis was performed to evaluate model performance. Survival analysis was conducted to verify the model's ability to predict prognosis in addition to the DCB (Fig. 1B). We describe the model construction procedure in the following section.

### TAN model robustly and interpretably predicted the DCB

We harnessed a BN graphical model to achieve accurate and interpretable predictions of the DCB. BNs graphically represent multivariate probability distributions (19) and are broadly applied in various biomedical tasks, including gene network feature selection (36), signaling network prediction (37), and hematological malignancy type prediction (38). Naïve Bayes (NB) networks are the simplest type of BNs but generally achieve favorable prediction accuracy (39). Based on Bayes’ theorem (Eq. 1), NB assumes that all covariates are equally important without distinction and are conditionally independent given a class value (Eq. 2) (39).


(1) $$p(C|X_{1},\ldots ,\;X_{n})=\frac{\;p(X_{1},\ldots ,\;X_{n}|C)p(C)}{\;p(X_{1},\ldots ,\;X_{n})}\Leftrightarrow \mathrm{Posterior}=\frac{\mathrm{Prior}\times \mathrm{Likelihood}}{\mathrm{Evidence}}.$$

(2) $$p(C|X_{1},\ldots ,\;X_{n})\propto p(C)\overset{n}{\underset{k=1}{\prod }}\;p(X_{k}|C).$$

The probability associated with a parent node (objective variable) is described as p(C), and the probability is updated to $p(C|X_{1},\ldots ,\;X_{n})$ when explanatory information from child nodes $\left(X_{1},\ldots ,\;X_{n}\right)$ is provided. In terms of its network structure, arrows (directed edges) extend from one node (a parent node or objective variable) to all other nodes (child nodes) (Fig. S1A). Despite its simple design and assumptions, NB achieves much better classification than expected and, therefore, is used in medical data analysis (40). Nonetheless, in its original form, it depends heavily on the assumption that the covariates are statistically independent, hampering its application to real-world biomedical data.

To address this, we utilized TAN models (Eq. 3):


(3) $$p(C|X_{1},\ldots ,\;X_{n})\propto p(C)\cdot p(X_{1}|C)\cdot p(X_{2}|X_{k},\;C)\cdots p(X_{n}|X_{l},\;C).$$

TAN alleviates the conditional independence between features, while keeping the directed acyclic graph (DAG) simpler than in conventional NN models (Fig. S1B and C). TAN does not assume conditional independence, partially allowing dependent relationships between variables (Eq. 2) (41). Therefore, since TAN can express a greater number of states, it must outperform NB models. Indeed, it has achieved high accuracy in numerous biomedical tasks, including risk stratification in pulmonary hypertension (42) and mammography (43). Here, we used NB and TAN to establish predictive models with higher accuracy and interpretability and compared their ability to predict the DCB in NSCLC patients.

**Fig. 2.**
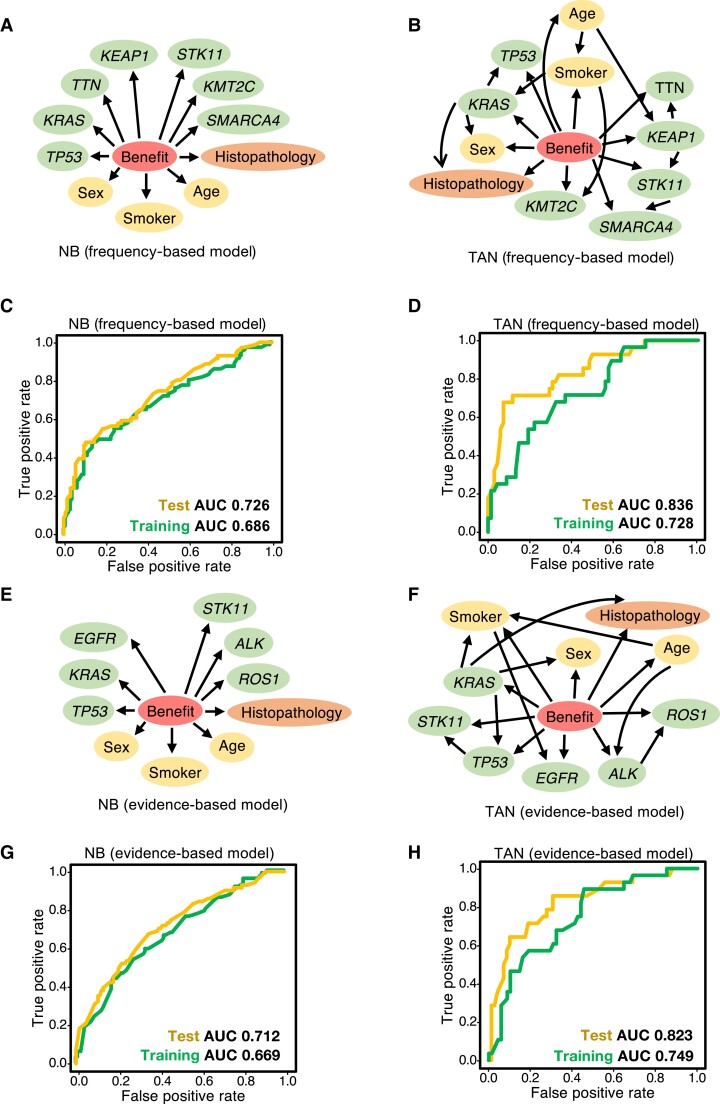
Bayesian network predicted the benefit of immunotherapy with high accuracy. A, B) The NB (A) and TAN (B) models were trained using the frequency-based data set. The predictor variable “benefit” (DCB, a central circle in each graph) is defined in the RECIST guideline (version 1.1) (22). Explanatory variables include patient data (sex, age, smoker), tumor tissue information (histopathology), and the frequency-gene data set (*TP53, KRAS, TTN, KEAP1, STK11, KMT2C, SMARCA4*). C, D) Predictive performance of the frequency-based models (A, B) for the test data set. TAN achieved greater accuracy than NB in terms of the AUC and was comparable with, or even more accurate than, state-of-the art methods (14, 44, 45). E, F) The NB (E) and TAN (F) models were trained using the evidence-based data set. G, H) Predictive performance of the evidence-based models (E, F) in the test data set. TAN achieved greater accuracy than NB in terms of AUC and was comparable with, or even more accurate than, state-of-the-art methods (14, 44, 45).

First, we constructed frequency-based models, using clinical data and the seven genes from the frequency-based geneset (*TP53*, *KRAS*, *TTN*, *KMT2C*, *SMARCA4*, *STK11*, and *KEAP1*) as covariates. The structure of frequency-based NB is shown in Fig. 2A. TAN structure was estimated using a training data set (Fig. 2B). For the NB model, the AUCs were 0.686 and 0.726 for the training and test data sets, respectively (Fig. 2C), and for the TAN model, 0.836 and 0.728, respectively (Fig. 2D). These results indicate that the TAN model has comparable or greater predictive accuracy than the NB model.

Next, we constructed evidence-based NB (Fig. 2E) and TAN (Fig. 2F) models using clinical information and the six genes from the evidence-based geneset (*KRAS*, *STK11*, *TP53*, *EGFR*, *ALK*, and *ROS1*) as covariates, using the same approach as with the frequency-based models. Using the test data set, the NB and TAN AUCs were 0.712 (Fig. 2G) and 0.823 (Fig. 2H), respectively, showing that TAN outperformed NB.

This demonstrates that the optimized TAN model outperforms NB and robustly predicts the DCB via frequency- and evidence-based approaches.

Its performance is comparable with that of other cutting-edge methods (14, 44, 45), while retaining explainability.

### Optimized TAN yields a robust graphical structure

We next evaluated the robustness of the structural estimation of our model in immunotherapy. We statistically generated multiple DAGs. Significant edges (internode connections) were detected when they appeared in >85% of the graphs.

**Fig. 3.**
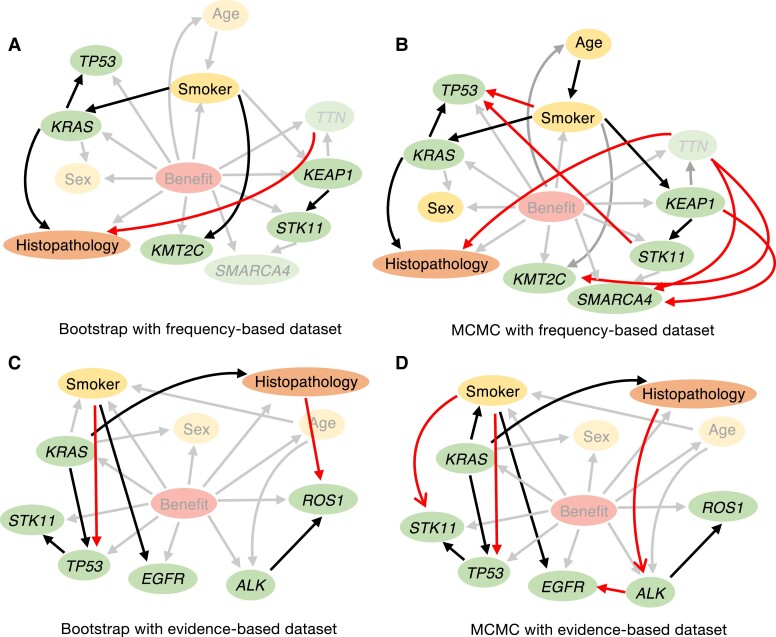
Evaluating the validity of the structure estimated by TAN through model-averaging methods. The linkages between nodes estimated by TAN were validated using model-averaging methods (bootstrap and MCMC). A, B) Bootstrap (A) and MCMC (B) sampling was used to create models using frequency-based data sets; connections considered to be significant in each process are illustrated. Relationships detected by the model-averaging methods but not by TAN are shown in red. Connections detected both by model-averaging methods and TAN are in black. C, D) Bootstrap (C) and MCMC (D) sampling were used to create models using evidence-based data sets; connections considered to be significant in each process are illustrated. The dependencies among variables estimated by TAN included many of the connections detected by model-averaging methods, indicating the robustness of our models. See also Tables 1 and 2.

We used two model-averaging methods (46, 47) to determine if the relationships identified by our methodology (Fig. 2B for the frequency-based model and Fig. 2F for the evidence-based model) were sufficiently robust. We applied bootstrap sampling (46) and Markov chain Monte Carlo (MCMC) methods to randomly constructed DAGs from a uniform distribution, as previously reported (47). This revealed several significant connections (Table 1, Fig. 3A and B for the frequency-based model; Table 2, Fig. 3C and D for the evidence-based model). These results demonstrate that model-averaging methods produce similar architectures, indicating that our method robustly discovered crucial relationships governing the immunotherapy response.

**Table 1.** Node verification via bootstrapping and MCMC for the frequency-based model.

Methods	Connection	Strength	TAN model
Bootstrap	Smoker-*KRAS*	0.956	○
	Smoker-*KMT2C*	0.874	○
	*TP53-KRAS*	0.990	○
	*KRAS*-cancer	0.968	○
	*TTN*-cancer	0.854	×
	*STK11-KEAP1*	1.000	○
MCMC	Cancer-*KRAS*	1.000	○
	Smoker-age	0.878	○
	Smoker-*TP53*	0.982	×
	Smoker-*KRAS*	0.982	○
	Smoker-*KEAP1*	0.962	○
	*KRAS-TP53*	1.000	○
	*TTN*-cancer	0.978	×
	*TTN-KMT2C*	0.868	×
	*TTN-SMARCA4*	0.974	×
	*STK11-TP53*	0.978	×
	*KEAP1-SMARCA4*	0.970	×
	*KEAP1-STK11*	1.000	○

**Table 2.** Node verification via bootstrapping and MCMC for the evidence-based model.

Methods	Connection	Strength	TAN model
Bootstrap	Cancer-*KRAS*	0.978	○
	Cancer-*ROS1*	0.880	×
	Smoker-*TP53*	0.942	×
	Smoker-*EGFR*	0.872	○
	*KRAS-TP53*	1.000	○
	*STK11-TP53*	0.847	○
	*ALK-ROS1*	0.956	○
Markov chain Monte Carlo	Smoker-*KRAS*	0.958	○
	Smoker-*STK11*	0.980	×
	Smoker-*TP53*	0.998	×
	Smoker-*EGFR*	0.908	○
	*KRAS-*cancer	1.000	○
	*KRAS-TP53*	1.000	○
	*STK11-TP53*	1.000	○
	*ALK-EGFR*	0.942	×
	*ROS1*-xancer	1.000	×
	*ROS1-ALK*	0.970	○

### Our TAN model accurately stratifies and predicts even with limited clinical information

**Fig. 4.**
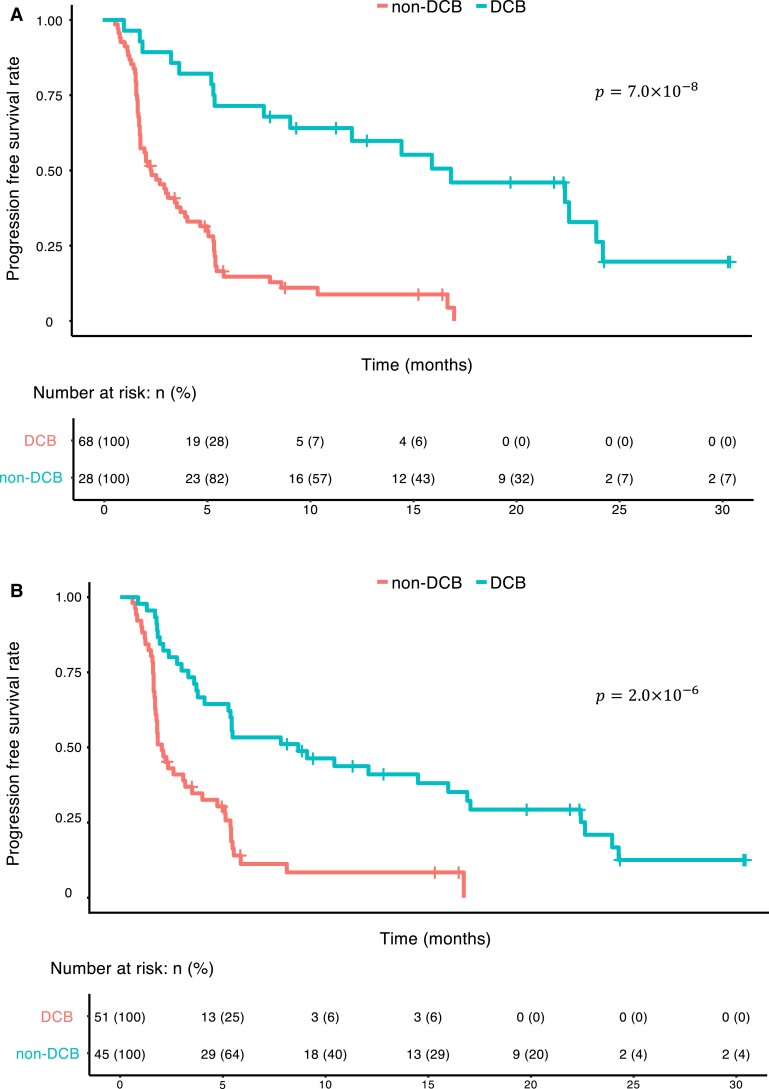
Our TAN-based interpretable models stratify NSCLC patient prognosis. A, B) We tested whether these TAN models are suitable for stratifying progression-free survival. We classified patients into two groups (“DCB” and “non-DCB”) based on the binary predictions of the frequency-based (A) or evidence-based (B) models and estimated progression-free survival status of the patients in our data set via the Kaplan–Meier method. The *P*-values were obtained through from log-rank tests.

Patient stratification is crucial to the development of personalized medicine (48). We thus evaluated our model's applicability to the stratification of NSCLC patients. We obtained the progression-free status of the patients in our data set from the cBioPortal database (7, 21). Our models identified two distinct and clinically significant groups based on binary prediction (Fig. 4A for the frequency-based model and Fig. 4B for the evidence-based model).

**Fig. 5.**
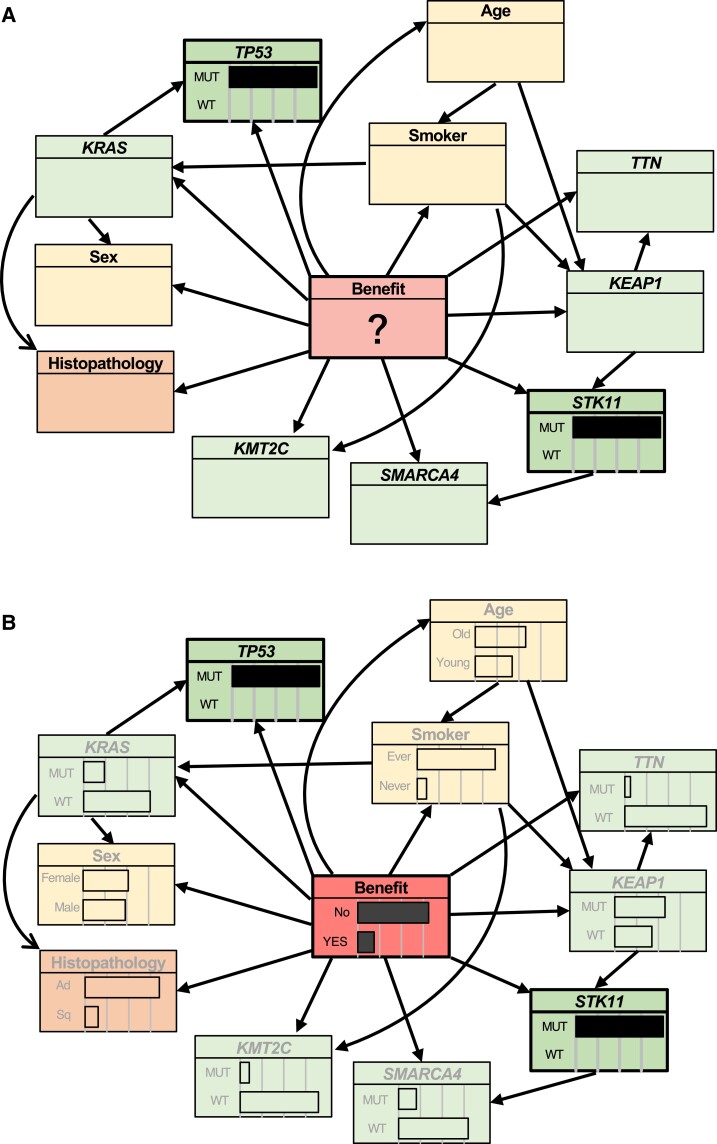
The optimized TAN model can infer the DCB even from limited data. A, B) We investigated whether our model (TAN, for instance) could infer the DCB from limited information. A) In this example, the only information provided to the model was the *TP53* and *STK11* variants in the patient. B) Using rejection sampling and approximate inference of the probability distribution of the unknown information, we were able to obtain probabilities for all of the hidden states. From only knowing that *TP53* and *STK11* were comutated, the model computed a response probability of 0.163, suggesting that immunotherapy would not be effective for this patient, which is consistent with the previous reports (26). The probability of unknown patient, genetic, histopathologic information and DCB status were calculated using our approach.

Importantly, the optimized TAN model can handle missing data and calculate conditional probabilities. For instance, it can predict whether a tumor will respond to immunotherapy, even if all that is known about a particular NSCLC patient is that they have *TP53* and *STK11* variants; the estimated response probability is 0.163, indicating that this patient would not benefit substantially from ICIs (Fig. 5). This speculation is consistent with established evidence (28). In contrast, for a young female patient with a *KMT2C* variant, but no *STK11* and *TP53* variants, the estimated ICI response probability is 0.592, indicating that ICI treatment would be valuable. This is important because previous models, including those based on ML and deep learning methods, cannot adequately handle missing data, requiring all of the necessary information (49). Given that data acquisition can be laborious, particularly in clinical settings, our model may help clinicians in decision-making, especially in situations with limited data.

### Our TAN models achieve better performance than cutting-edge ML methods

We attempted to compare our method with those of recent reports. Ouyang et al. (45) repeated a univariate cox proportional hazards analysis for each of the genes associated with hypoxia, immunity, and epithelial–mesenchymal transition and selected the significant variables (genes). Then, they constructed a Lasso regression model on the risk score defined by the coefficient in the hazards analysis multiplied by the gene expression (∑coefficient×geneexpression). Based on this model, they predicted variables that represented responsiveness. Thus, we followed this procedure using our data set. We used the data set from Rizvi et al. (7) to perform univariate cox proportional hazards regression and selected significant (*P* < 0.05) variables (Table S2). Next, we created a model with 10 cross-validations in the same way and evaluated the prediction accuracy for the DCB. In addition, predictions were made using data from Hellmann et al. (21) to ensure robustness to external cohort data. The AUCs were 0.611 and 0.622 for the training and test data, respectively [from Rizvi et al. (7)], and 0.523 for the external cohort data [from Hellman et al. (21)] (Fig. S2A). Thus, we concluded that the accuracy achieved by our method was higher than that of regression analysis (45) and robust to external cohort data.

We also compared our approach with that of deep learning-based models. Tian et al. (14) and He et al. (44) developed convolutional NNs and used computed tomography images as input. However, since our clinical data were in tabular form, not as images, we could not use the exact same method. Instead, we applied a relatively simpler architecture named multilayer perceptron to evaluate the performance of the NN in our data set (Fig. S1C). Using the genes selected in the frequency model, we trained a multilayer perceptron on our data set. The AUC was 0.925 for the training data and 0.588 for the test data (Fig. S2B). We also trained another NN using the genes selected in the evidence-based model, and its AUC was 0.857 for the training data and 0.585 for the test data (Fig. S2C). Thus, our TAN-based methods performed better than NN based methods in terms of accuracy without overfitting.

### Our approach is robust with an external data set

Finally, to further support the robustness of our approach, we used different data sets for training/testing a new TAN model. Specifically, we used the cohort data of Rizvi et al. (7) for training/testing and the cohort data from Hellman et al. (21) for evaluating the external validity of the model. However, the cohort data from Rizvi et al. only included adenocarcinoma, whereas that from Hellman et al. also included other subtypes. Therefore, we limited our analysis to lung adenocarcinoma patients and used the cohort from Rizvi et al. in model reconstruction and the cohort from Hellman et al. as external data. *TP53*, *PTPRD*, *SMARCA4*, *PTPRT*, *KMT2C*, *KRAS*, *STK11*, *KEAP1*, *EPHA3*, *EGFR*, and *RBM10* were the most frequently mutated genes (>10%) in the training cohort and were used to build the new TAN model (Fig. S3A). The AUCs for the frequency-based model were 0.841 for the training data and 0.732 for the test data. The AUC for the external validation cohort was 0.740 (Fig. S4A), which shows the generalizability of our approach.

Meanwhile, the AUCs for the evidence-based model (Fig. S3B) were 0.792 for the training data and 0.770 for the test data. The AUC for the external validation cohort was 0.635 (Fig. S4B). The reason behind this might be that clinical knowledge for overall lung cancer was not appropriate to this adenocarcinoma-specific subpopulation. Still, the evidence-based model was more accurate than those from previous reports (Fig. S2) (14, 44, 45).

## Discussion

Most prior attempts to predict immunotherapy responses have used ML-based approaches (13–15), which are complex “black-box” systems that cannot handle missing data. Moreover, they require all the clinical and molecular information to be provided as input. However, such data are often difficult to obtain, especially in hospitals with limited resources, which hampers the clinical application of these models. In addition, transparency and clinical validation are necessary to achieve reliable medical AI (16). Therefore, we sought to develop an interpretable and robust model that predicts NSCLC patient responses to immunotherapy. We used clinical information, selected genetic variant data based on frequency- and evidence-based approaches, and established optimized TAN models. Our approach is comparable with, or even superior to, several cutting-edge ML methods (14, 44, 45), while retaining explainability. It provides clinically informative predictions even when data are limited (Fig. 5), as this is quite common in clinical settings. Furthermore, because this model only computes conditional probabilities based on Bayes’ theorem (46), it is possible, if necessary, to control which nodes should (or should not) have connections, using a “white list” (or “black list”) based on expert knowledge. Our models could also be used to generate hypotheses for future research. For instance, our inferences based on limited data (Fig. 5) are consistent with the findings of recent reports (28, 30). This suggests that, by using more clinical samples with diverse genetic profiles, our approach may reveal new therapeutic targets, providing an invaluable resource for both clinical and basic medicine.

We surveyed data sets from cBioPortal and found three cohorts whose target patient population was advanced lung cancer (7, 21, 50). Then, we adopted the two (7, 21) that provided DCB data. One included only lung adenocarcinoma data (21), while the other one also included lung squamous cell carcinoma data (7). Therefore, we combined the two in our analysis to examine more pathological profiles. We selected several genes based on genetic variant frequency or previous evidence: *KRAS*, an immunomodulatory oncogenic gene, leads to escape from immunotherapy (29); together with *TP53* or *STK11* variants, *KRAS* variants are a potent prognostic factor (28, 31). Moreover, *STK11* is associated with diminished immunotherapy response (30); and *BRAF* variants, which are associated with a higher tumor burden, may make tumors vulnerable to immunotherapy (51). Lastly, genetic variants in driver oncogenes such as *EGFR*, *ALK*, and *ROS1* in tumors cause a lack of immunogenicity and, thus, a poor response to immunotherapy, regardless of PD-L1 score (52).

Both frequency- and evidence-based models predicted the target variable, DCB, with comparable or even better metrics than several state-of-the-art methods (14, 44, 45), and the estimated edges between the variables were consistent with previous reports. Generally, prior knowledge to construct a network structure is limited. Therefore, between the two methods, especially in clinical use, a frequency-based model would be the better choice because it could learn solely from data.

It should be noted that most ML methods involve updating parameters for the purpose of error minimization. However, the learning target of a BN is generally its structure and is estimated with the chi-square test (53) or information criteria (54). Given that overfitting was not part of the process to minimize errors, the performance on test data could exceed that on training data.

Consistent with an earlier analysis of clinical data on the utility of TAN (43), our TAN-based approach provided greater value than the NB model. Due to the small sample size, the conventional NB model using hill-climbing methods was unable to construct suitable structures for inference (Fig. S5), suggesting that our approach is better suited for inference with small data sets. Furthermore, TAN alleviates the conditional constraints imposed by NB. Here, some of the essential connections in TAN structural learning were also detected via model-averaging using bootstrap sampling and MCMC (Fig. 3). For instance, our model-averaging findings obtained using the frequency-based approach (Fig. 3A and B) strongly suggested an association between smoking status and *KRAS* variants, which has been reported previously (32). There are also several gene-related relationships reported as important prognostic factors, such as covariants of *STK11* and *KEAP1* (55) and *KRAS* and *TP53* (56). In addition, associations between histopathology and *KRAS* (57) or *TTN* (58) have also been discovered. Other strong connections between nodes inferred with model-averaging methods (Fig. 3) are expected to reveal unknown immunotherapy-related relationships.

Determining the direction of causality from data alone remains highly challenging, especially in high-dimensional data (59). Therefore, the direction of the arrow in our models is chosen at random, and we limit our assertions only to the fact that relationships are relevant. We believe that the direction of causality should be ensured via high-quality studies such as randomized control trials. A second limitation is that although we used well-known risk factors in our model, such as sex (24, 25), it is still missing some others such as exposure to asbestos, radiation, secondhand smoke, history of pulmonary fibrosis, and alcohol consumption. We could not include these because the information was not provided in the data set, but we are sure they would be of great value in future research. Another limitation is that selection bias cannot be ruled out due to the integrated use of public data sets. Although the data sets comprised patients who underwent immunotherapy, it is plausible that the data did not represent a specific population. In addition, the strength of the internode relationships that we estimated may reflect the small sample size, and an analysis employing a larger data set may reveal additional relationships. Therefore, we have developed a web-based intuitive DCB estimator (https://pred-nsclc-ici-bayesian.shinyapps.io/Bayesian-NSCLC/) that does not require computational expertise. Future analyses with larger clinical samples are likely to overcome these limitations and provide further support for the validity of this approach.

In summary, our robust TAN models are comparable with, or even superior to, other predictive models for immunotherapy. They can predict meaningful and interpretable connections and inferences, even with a limited number of observations. We hope that this model will guide clinicians in selecting NSCLC patients who require immunotherapy and expect it to be easily applied to other types of cancer.

## Materials and methods

### Public cohorts

The cBioPortal (http://www.cbioportal.org) (20) was accessed to retrieve clinical and genetic variant data for NSCLC patients. We chose two studies examining the effects of ICIs on NSCLC patients (7, 21) to use as the data set for this study. The inclusion criteria and clinical and genetic information for the two cohorts are explained in the original papers (7, 21).

The characteristics of our data set are shown in Table 1, including age (<65 years or not), sex, smoking status, and histopathological information. We excluded 25 samples, comprising mostly of those with unspecified histological data (described only as “NSCLC”) and a few categorized as “large cell neuroendocrine carcinoma.” We obtained data on genetic variants to prepare the “frequency-based” and “evidence-based” genesets. We also analyzed the progression-free status of the patients in these cohorts.

### Model construction

The foundation of TAN lies in the structural constraint that each explanatory variable can be connected with one node other than an objective variable.

Thus, a complete undirected graph with nodes and edges is constructed to estimate this structure. In this stage, one node is wholly connected to every other node. Each variable is described as $X_{1},\ldots ,X_{n}$, and mutual information values are given to each edge. The edge weights between two nodes $\left(X_{i},\;X_{j}\right)$ are given by Eq. 4:


(4) $$I(X_{i},\;X_{j}|C)=p(X_{i},\;X_{j},\;C)\log\frac{\;p(X_{i},\;X_{j}|C)}{\;p(X_{i}|C)p(X_{j}|C)}.$$

To obtain the constrained structure of TAN from this complete graph, a structure with the highest total weights under the constraint was used as an estimated structure. To transform the given undirected graph tree into a directed one, a root variable was randomly chosen, and the direction of the edges is set to outward from the root variable (19, 60, 61). Then, the data were randomly split into training (2/3) and test data (1/3) (Fig. 1B). In addition, cross-validation was not employed, and the split was performed once as in another study (62). Given that the model-averaging method is for assessing edge validity (46, 60), it was not used to test predictive performance.

The training data were used to construct the models and to learn the conditional probability between each node. ROC curves were constructed from the test data predictions. The model was constructed with the bnlearn (4.7.1) R package and evaluated with the ROCR package (1.0-11).

### Model evaluation

Model-averaging methods were adopted to measure the reliability of the connections between nodes in the network by performing multiple structural estimations using the hill-climbing method (60). In the BN, it is important to measure the confidence level for a particular graph feature (the graph edge). This confidence level (in terms of relative frequencies), referred to as arc strength (46, 60, 63), is defined as the number of times an internode connection appears while generating multiple graphs; frequencies >85% are considered strong (60).

Two model-averaging methods were adopted for evaluating the node connections of our model. The first is the bootstrap approach, which applies nonparametric bootstrapping to generate multiple networks and estimates the arc strength (46, 60).

Algorithm 1 provides the specific method.

**Table:** 

Algorithm 1
1. For b=1,2,…,B:
1.1. Sample a data set $D_{b}$ from the original data *D* via nonparametric bootstrapping.
1.2. Learn the BN, $g_{b}=(V,\;E_{b})$ from $D_{b}$.
2. Estimate the arc strength, defined as follows:
$\hat{\;p_{i}}=\hat{P}(a_{i})=\frac{1}{B}\overset{B}{\underset{b=1}{\sum }}\;N_{\{e_{i}\in E_{b}\},}$
where N is equal to 1 if $e_{i}\in E_{b}$ and 0 otherwise.

The second model-averaging method is the random generation of multiple graphs from a uniform distribution using the MCMC algorithm (Algorithm 2). One graph was randomly sampled for every 50 graphs generated, and the arc strength from 500 sampled graphs was measured (63).

**Table:** 

Algorithm 2
1. For b=1,2,…,B:
1.1. Sample a data set $D_{b}$ from the original data *D* via parametric or nonparametric bootstrapping.
1.2. Learn the BN, $g_{b}=(V,\;E_{b})$ from $D_{b}$.
2. Estimate the arc strength, defined as follows:
$\hat{\;p_{i}}=E(e_{i}|D)\approx \frac{1}{B}\overset{B}{\underset{b=1}{\sum }}\;N_{\left\{e_{i}\in E_{b}\right\}}P(g_{b}|D).$

The robustness of the TAN structure estimation was evaluated by examining whether the connections between nodes determined to be significant by these model-averaging methods were also present in the TAN structure.

### Inference with limited evidence

To estimate the conditional probability of an event using only the limited evidence available, the cpquery function of the bnlearn package (4.7.1) was used. In this method, logic sampling, or an approximate inference, enables it to obtain the probability (64). First, a new data set is created by randomly extracting data that match the specified evidence from the whole data set. In our case, patient profiles and genetic variant information were specified. By repeating this method, 1 million random samples were generated, and an approximate probability was returned based on them.

### Survival analysis

Survival analysis was conducted using the survival package (3.3-1). *P* < 0.05 was considered statistically significant.

## Supplementary Material

pgad133_Supplementary_Data

## Data Availability

All clinical and genetic information is available from the cBioPortal database, (http://www.cbioportal.org), and the specific explanation of each cohort can be obtained in the original papers (7, 21). A web application (https://pred-nsclc-ici-bayesian.shinyapps.io/Bayesian-NSCLC/) using the shiny package (1.7.2) provides both frequency- and evidence-based models. The R code for training the NB and TAN models and for validation and scoring via ROC and survival analysis is available at GitHub (https://github.com/Hideki-Hozumi/Bayesian-Network-NSCLC).
