# Supplementary material for: Bayesian network enables interpretable and state-of-the-art prediction of immunotherapy responses in cancer patients
Source: PNAS Nexus. 2023 Apr 13;2(5):pgad133. doi: 10.1093/pnasnexus/pgad133 (PMC10162686; doi:10.1093/pnasnexus/pgad133)
Supplement: pgad133_Supplementary_Data [file pgad133_supplementary_data.docx]

**SUPPLEMENTARY MATERIAL**

**Bayesian network enables interpretable and state-of-the-art prediction of immunotherapy responses in cancer patients**

Hideki Hozumi,^1^ Hideyuki Shimizu^2,*^

^1^Keio University School of Medicine, Tokyo 160-8582, Japan.

^2^Department of AI Systems Medicine, M&D Data Science Center, Tokyo Medical and Dental University, Tokyo 113-8510, Japan

^*^Corresponding author:

Hideyuki Shimizu

[h_shimizu.dsc@tmd.ac.jp](mailto:h_shimizu.dsc@tmd.ac.jp)

**Supplementary Tables**

**Table S1. Characteristics of the dataset**

| **Characteristic** | **Number** |
| --- | --- |
| **Age** |  |
| ≧65 | 157 |
| <65 | 133 |
| **Histopathology** |  |
| Adenocarcinoma | 256 |
| Squamous | 34 |
| **Sex** |  |
| Male | 141 |
| Female | 149 |
| **Smoker** |  |
| Ever | 231 |
| Never | 59 |
| **Durable clinical benefit** |  |
| Yes | 99 |
| No | 191 |
| ***TP53*** |  |
| Genetic variant | 168 |
| Wild type | 122 |
| ***KRAS*** |  |
| Genetic variant | 100 |
| Wild type | 190 |
| ***STK11*** |  |
| Genetic variant | 50 |
| Wild type | 240 |
| ***KEAP1*** |  |
| Genetic variant | 47 |
| Wild type | 243 |
| ***TTN*** |  |
| Genetic variant | 34 |
| Wild type | 256 |
| ***KMT2C*** |  |
| Genetic variant | 32 |
| Wild type | 258 |
| ***SMARCA4*** |  |
| Genetic variant | 30 |
| Wild type | 260 |
| ***EGFR*** |  |
| Genetic variant | 26 |
| Wild type | 264 |
| ***ALK*** |  |
| Genetic variant | 13 |
| Wild type | 277 |
| ***ROS1*** |  |
| Genetic variant | 7 |
| Wild type | 283 |

**Table S2. Variable selection using the method by Ouyang et al.**

| **Gene** | **p-value** |
| --- | --- |
| *TP53* | 0.02070419 |
| *ATRX* | 0.02141156 |
| *JAK1* | 0.04571599 |
| *EGFR* | 0.04033987 |
| *ARID1B* | 0.04992846 |
| *POLE* | 0.04393400 |
| *RHOA* | 0.04437944 |
| *FH* | 0.00300668 |
| *SUFU* | 0.01182486 |

**Supplementary Figures**

**
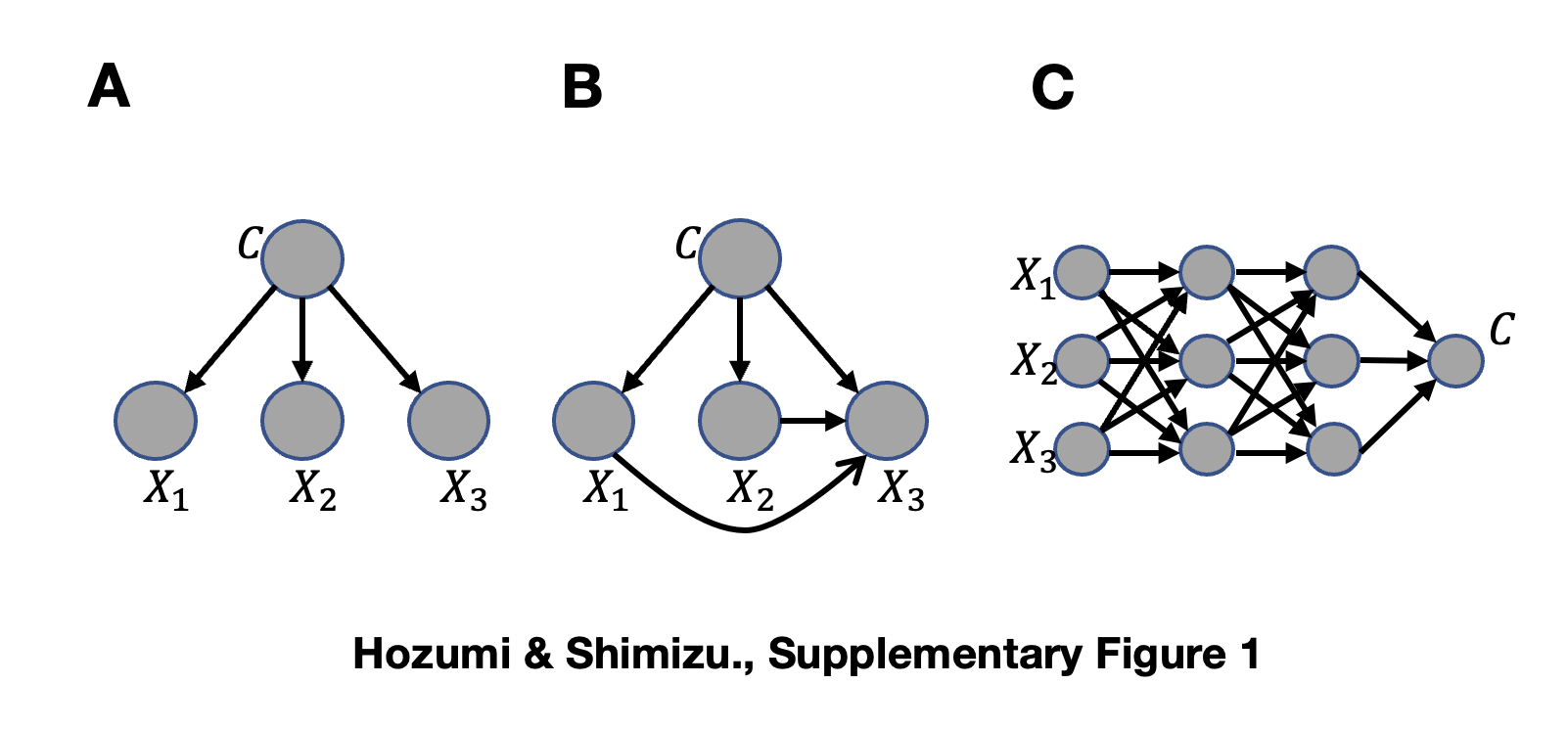
**

**Figure S1. Structures of the naïve Bayes (NB) model, tree-augmented naïve Bayes (TAN) model, and a neural network**

(**A**) NB model: the node (an objective variable, indicated by C) is directly connected with covariates (indicated by $X_{1}, X_{2}, X_{3}$). (**B**) TAN: unlike the NB model, an additional node can be connected between nodes. (**C**) Neural network: every node has connections with all other nodes in the previous layers. This illustrates that, based on their architecture, the NB and TAN models differ substantially from the neural network in their constraints.

**
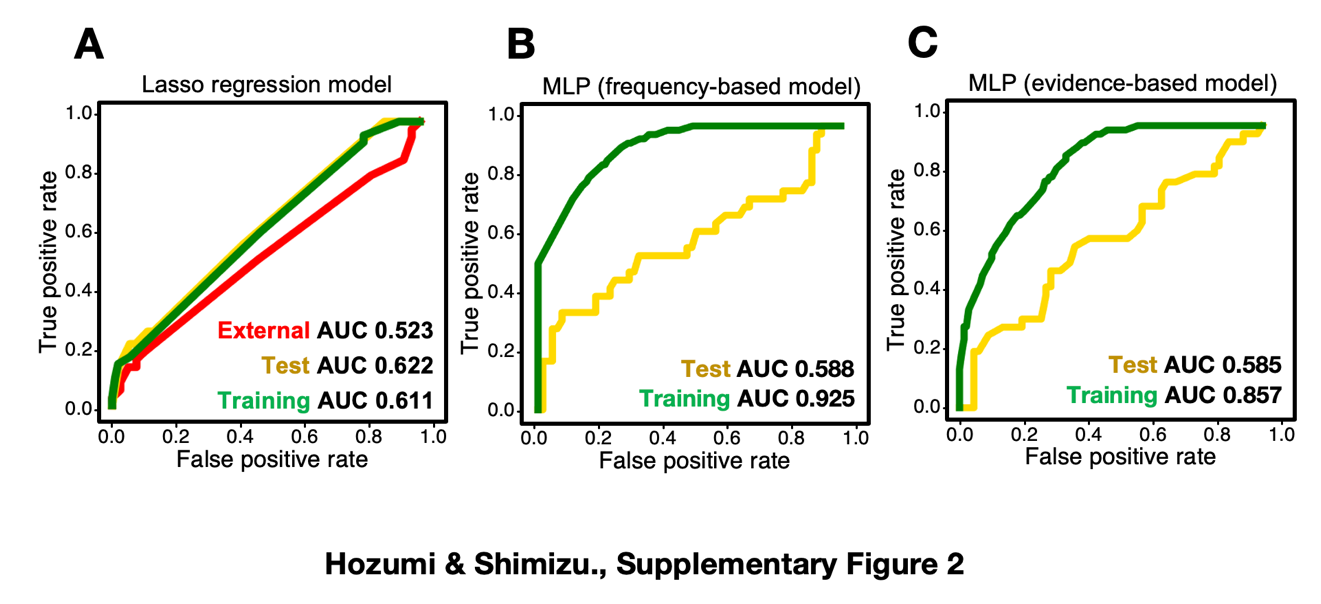
**

**Figure S2. Comparison to other machine learning methods**

(**A**) Performances of Lasso regression model (45) to data from Rizvi et al. (7) (Train/Test) and from an external cohort (21). (**B, C**) Performances of multilayer perceptron (MLP) models to data from Rizvi et al. (7) and Hellmann et al. (21).

**
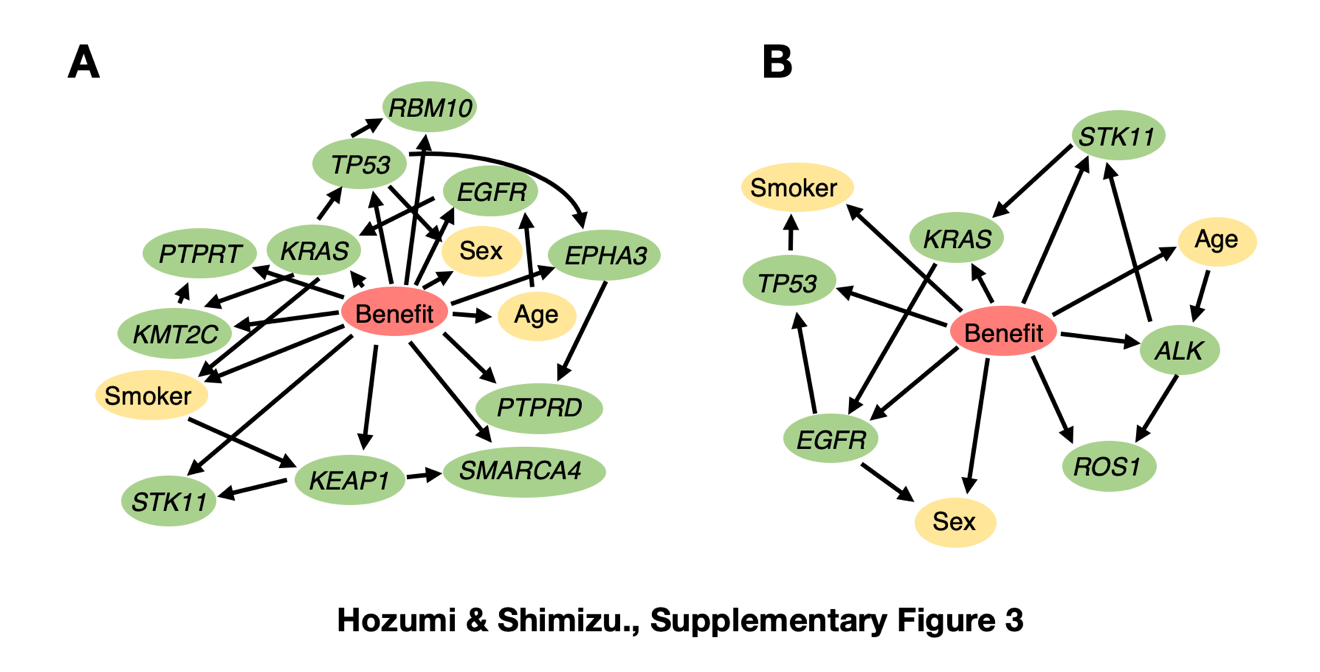
**

**Figure S3. Proposed architecture for the data from Rizvi et al.**

We created tree-augmented naïve Bayes models using data from Rizvi et al. (7) for frequency-based (**A**) and evidence-based (**B**) gene selection methods. Explanatory variables include patient data (yellow) and genetic information (green).


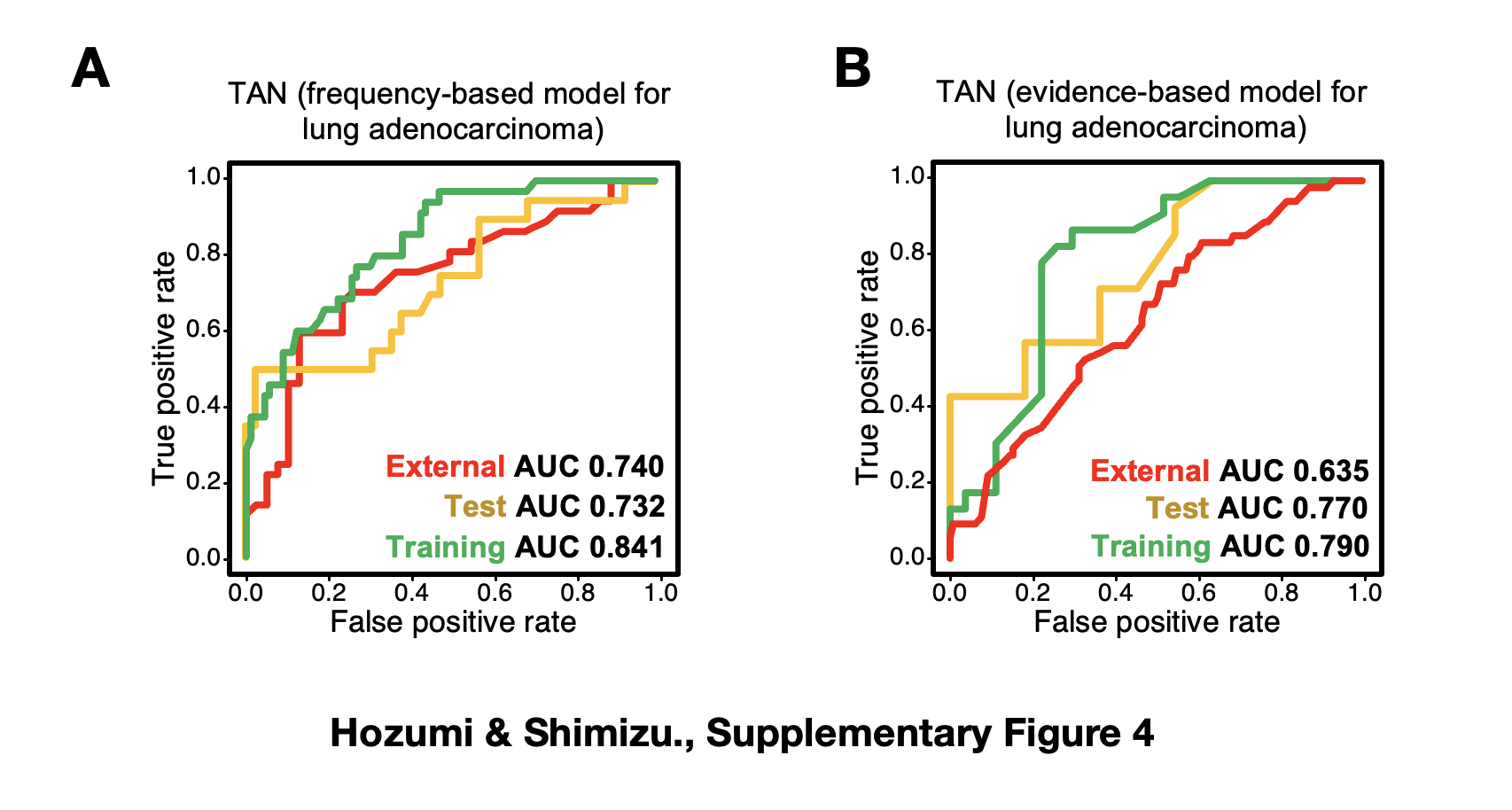


**Figure S4. Our approach is robust with an external dataset**

(**A**) Receiver operating characteristic (ROC) curve for the frequency-based tree-augmented naïve Bayes (TAN) model shown in Supplementary Figure 4A to train/test data from Rizvi et al. (7) and an external cohort (21). (**B**) ROC curve for the evidence-based TAN model shown in Supplementary Figure 4B to train/test data from Rizvi et al. (7) and an external cohort (21).


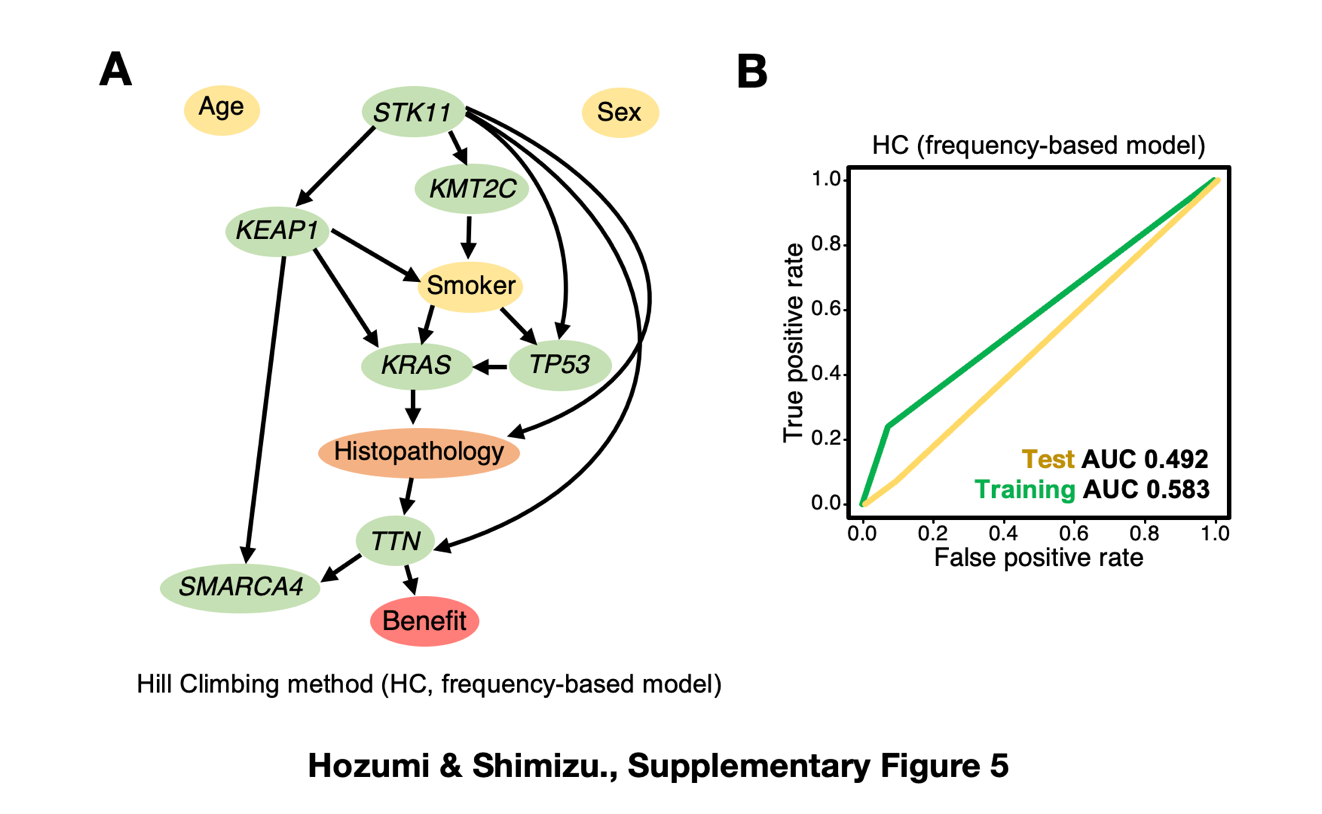


**Figure S5. A constraint-free Bayesian network did not achieve better performance**

(**A**) Network structure estimated using an unconstrained Bayesian network model and the hill-climbing method. Note that not all variables are connected with edges. (**B**) The unconstrained Bayesian network model in panel (**A**) performed worse, in terms of area under the curve (AUC), than the naïve Bayes (NB) and tree-augmented naïve Bayes (TAN) models. For the NB model AUC values, see Figures 2C and 2G; for those of the TAN model, see Figures 2D and 2H.
